# Effect of the irregular shelterwood system on soil organic carbon stock and soil quality of *Shorea robusta* Gaertn. f. forest in Nepal

**DOI:** 10.1016/j.heliyon.2024.e35441

**Published:** 2024-08-04

**Authors:** Anil Poudel, Santosh Ayer, Rajeev Joshi, Jeetendra Gautam, Sachin Timilsina, Keshav Khadka, Kishor Prasad Bhatta, Menuka Maharjan

**Affiliations:** a College of Natural Resource Management (CNRM), Agriculture and Forestry University, Katari, 56310, Nepal; b Faculty of Forestry, Agriculture and Forestry University, Hetauda, 44100, Nepal; c Institute of Forestry, Tribhuvan University, Pokhara, 33700, Nepal; d Ministry of Forests, Environment and Soil Conservation, Lumbini Province, 32900, Nepal; e Research and Development Centre (RDC), Kathmandu, 44600, Nepal; f School of Forestry and Natural Resource Management, Institute of Forestry, Tribhuvan University, Kathmandu, 44600, Nepal; g Institute of Forestry, Tribhuvan University, Hetauda, 44100, Nepal

**Keywords:** *Irregular shelterwood system*, *Soil properties*, *Soil quality index*, *Forest management*

## Abstract

The effective management of forests relies on the crucial role played bysilvicultural systems. However there exist a significant knowledge gap regarding impact of these systems in Nepalese forests. Therefore, this research was conducted to assess the effects of the forest management activities under irregular shelterwood system on soil organic carbon (SOC) stock and the overall soil quality of Sal (*Shorea robusta* Gaertn. f.) forests in Terai region of Nepal. Stratified random sampling method with 1.67 % sampling intensity was adopted in this study where management of stands was used as basis of strata. A total of 30 composite soil samples (15 each from managed and unmanaged forest stands) were collected from a depth of 0–30 cm, taken from the four corners and the center of each plot. Soil quality index (SQI) method was used for soil quality assessment using indicators on the basis of prior studies conducted in Nepal. Our study found significant difference in soil parameters except organic carbon, pH, silt, and clay among the managed and unmanaged forest stands (p < 0.05). SOC stock of unmanaged forest stands (48.87 ± 1.34 ton ha^−1^) was significantly greater than managed forest stands (27.76 ± 1.27 ton ha^−1^). Similarly, unmanaged forest stands demonstrated better soil quality with higher SQI value (0.66) than managed forest stands (0.50). This negative impact of irregular shelterwood silviculture system highlights the necessity for management interventions to enhance SOC stock and overall soil quality. To establish a robust conclusion, further replication of similar studies at different soil depths and in other management regimes, along with longitudinal studies, is essential.

## 1 Introduction

*Shorea robusta* (Family: Dipterocarpaceae), commonly known as Sal, is a commercially and ecologically significant tree species native to Nepal's Terai area [1,2]. Out of the total forest area of 6.61 million hectares in Nepal, which constitutes 44.74 % of the country's land area, Sal forest accounts for 15.27 % of this forest area [3]. Sal forests in Nepal are vital ecological and economic resources, closely connected to the nation's identity and prosperity [3,4]. Predominantly found in the Terai region, these forests have historically served multiple roles, significantly contributing to biodiversity conservation, timber production, and the livelihoods of local communities [5]. Their substantial ecological importance extends beyond Nepal's borders, as they are part of the larger Terai-Duar savanna and grasslands ecoregion, a biodiversity hotspot that supports diverse plant and animal species [6]. However, the sustainability of Sal forests is under threat due to several challenges, including aging trees exhibiting hollowness, disease, over-maturity, and poor health as a result of historical protective management practices [[7], [8], [9]]. These issues are further compounded by the increasing human population and rising demands for timber and non-timber forest products [10]. Since the 1980s, community-based forest conservation has been implemented to protect forest land from deforestation and degradation [11]. These community-based forestry programs have not only aimed to reverse deforestation and degradation but also to promote afforestation and reforestation efforts [12]. Despite the success of participatory forest management in safeguarding forest lands, the focus on forest stand quality, productive potential, and biodiversity was often overlooked [13,14]. Recognizing the multifaceted functions of forested landscapes, including their roles in ecosystem functioning, biodiversity conservation, and socio-economic development, the government of Nepal introduced Scientific Forest Management in both community-managed and government-managed forests [15].

Scientific Forest Management is an approach promoted by the Government of Nepal that emphasizes the application of appropriate silviculture systems and forest management principles [16]. This method involves the design of systematic compartments with a fixed rotation age, primarily following the shelterwood silviculture system [9,17]. Irregular sheterwood system is most preferred silvicultural system in the Sal forests in Terai region of Nepal [15,[17], [18], [19]] with the goal of establishing desirable species with a prolonged regeneration period than a regular system [20,21]. The irregular shelterwood system is a silviculture method where most trees are removed during felling, except for a few mother/shelter trees (15–25 per hactare), while poles are preserved as advanced crops, resulting in an uneven crop composition [16,22]. While this system has been found beneficial in promoting regeneration, it can negatively affect species biodiversity by favoring the regeneration of dominant species like Sal, thereby reducing the diversity of other species that require different light or soil conditions [9,15,[17], [18], [19]].

Soil carbon stock is a vital component of forest ecosystems, playing a significant role in the global carbon cycle and climate regulation. Forest soils act as major carbon sinks, sequestering large amounts of carbon dioxide from the atmosphere [23]. Estimates suggest that global soil organic carbon stores are roughly 1500 Pg of carbon (Pg C), which is two-to-three times the amount of carbon present in the atmosphere and exceeds the carbon stored in plant biomass across most IPCC climatic regions [24]. Both natural and anthropogenic disturbances impact soil carbon stocks by influencing the rates of organic matter input and decomposition. Natural disturbances, such as wildfires, pests, diseases, and windthrow, can temporarily reduce soil carbon stocks in forests [25,26]. Human activities, including the conversion of forests to other land uses and modifications of forests for the provision of forest products and services, also affect forest soil carbon stocks [27,28]. The rate and direction of soil carbon changes depend on previous land use, soil type, climate, tree species, forest age, and management practices [29]. Active management of forests allows for better control of carbon stocks because forest dynamics are less influenced by natural disturbances [28]. Some studies have found an initial decline in soil carbon following management activities, but over time, soil carbon levels may increase as the ecosystem stabilizes [[30], [31], [32]]. Therefore, both short-term and long-term studies are necessary to fully understand these dynamics and their implications for carbon sequestration and climate change mitigation.

Soil quality, encompassing physical, chemical, and biological properties, fundamentally determines the soil's ability to sustain life and support productive ecosystems. Key indicators such as soil structure, nutrient availability including phosphorus and potassium, soil organic carbon, bulk density, pH levels, and soil texture are critical in assessing soil health [[33], [34], [35]]. Different forest management practices can have diverse effects on soil quality. For instance, intensive practices like clearcutting can lead to severe soil disturbance, compaction, and loss of organic matter, thereby affecting soil structure negatively and reducing water infiltration [36]. In contrast, selective logging, if practiced carefully, may mitigate some impacts by retaining certain trees and organic inputs, which can help maintain soil structure and nutrient cycling to some extent [37]. While thinning can enhance light and nutrient availability for remaining trees, it may temporarily increase soil compaction due to machinery use but often leads to less severe impacts compared to clearcutting [38]. Similarly, removing understory vegetation may reduce organic matter input and affect microbial activity in soil [38]. Land managed as agroforestry systems in various regions have demonstrated improvements in soil fertility due to enhanced nutrient availability and microbial activity facilitated by organic inputs from tree litter and root turnover [39,40]. Studies across different forest types and regions highlight varied effects of these management practices on soil quality. For example, research in various temperate and tropical forests has shown that soil pH and nutrient availability can be influenced by the type and intensity of forest management [[41], [42], [43]]. Practices that retain organic matter, such as leaving slash or using low-impact harvesting techniques, generally support better soil health by preserving soil structure and nutrient cycling capabilities [44,45]. Understanding the impacts on soil quality due to different management modalities is crucial for adapting forest management strategies that promote soil resilience and long-term productivity. However soil quality cannot be measured directly but can be inferred by measuring soil physical, chemical, and biological properties that serve as quality indicators [[46], [47], [48]]. One of the most common quantitative approaches for soil quality assessment is to adopt soil quality indices (SQIs) [[49], [50], [51]]. SQI is an integrated approach that combines multiple soil property measurements into a single index to provide an overall assessment of soil quality [51]. Different approaches exist for estimating SQI, including additive systems based on common soil parameters [52,53], soil fertility/nutrient/index approaches [54,55], and statistical model-based SQI [51]. Among them, Abdu et al. [51] recommended an additive system based on common soil parameters method to compute SQI due to its consistent outcomes for soil quality assessment.

Several previous research have showed effects of shelterwood silvicultural system on soil properties in short term and long term. For instance, Elliot and Knoepp [56] found reduced extractable calcium, magnesium, potassium, cation exchange capacity, pH, bulk density, A-horizon depth, total carbon, and nitrogen in the southern Appalachians under irregular shelterwood systems [56]. Similarly, Klein et al. [57] reported short-term decrease in soil organic carbon due to intensive shelterwood cuts in Nothofagus pumilio forests of the Chilean Patagonia. A study by Christophel et al. [58] observed considerable long-term decrease in organic carbon and nitrogen stocks in shallow calcareous forest soils of the Bavarian Alps under different shelterwood systems. Recent study by Carpenter et al. [59] found that irregular shelterwood harvests initially lowered nitrogen, potassium, phosphorus, and organic carbon in a temperate oak hardwood forest in the United States. However these differences were recovered over time, with no significant differences in soil nutrients by 11–15 years post-timber harvest [59]. Despite such evidences regarding the implications of shelterwood system on forest soil, it is still overlooked in Nepal. Majority of studies in Nepal are concentrated on how irregular shelterwood system is affecting forest structure, composition, regeneration, and tree diversity [17,19,60,61]. A recent study by Aryal et al. [9] also included soil properties to study effect of irregular shelterwood system in a collaborative forest of central lowlands of Nepal [9]. However, there is a notable gap in studies specifically addressing the impacts of the irregular shelterwood system on soil quality in Sal forests in Nepal. Therefore, the major objectives of this study is to assess impact of irregular shelterwood system on i) soil organic carbon (SOC) stock and ii) soil quality between managed and unmanaged forest stands of community managed Sal forest. We hypothesize that the application of the irregular shelterwood system in the forest has a significant impact on SOC stock and soil quality. Findings from this study will provide valuable suggestions to forest managers regarding management of Sal Forest soil under irregular shelterwood system.

## 2 Materials and methods

### 2.1 Study area

**Fig. 1:**
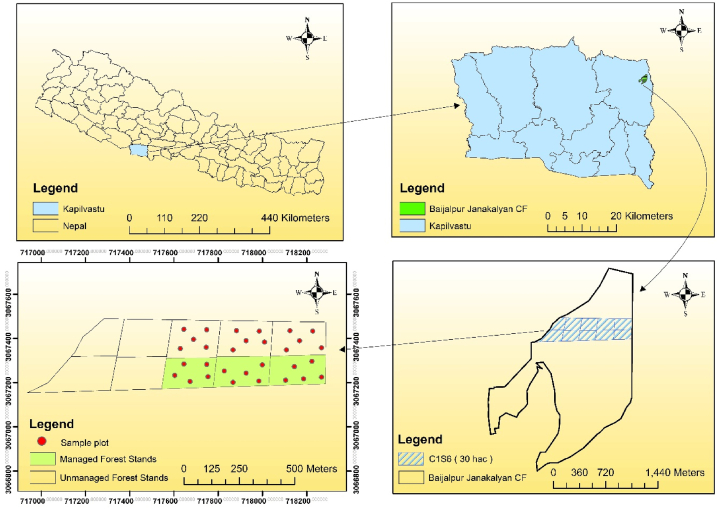
Location map of study area.

This study was carried out in Baijalpur Janakalyan Community Forest which is situated in Banganga Municipality-2 of Kapilvastu district in Lumbini Province, Nepal (Fig. 1). The community forest spans a total area of 263.43 hactares (ha), with a productive area of 239.47 ha consisting predominantly of natural forest. Situated in the Terai region, the forest experiences a tropical climate with an average annual precipitation of 1870 mm and temperature of 21.5 °C. The forest features a canopy cover of 70 % and is characterized by gentle slopes (0–15°). The elevation varies between 130 and 150 m above sea level, and the terrain is predominantly flat. The major tree species found in this community forest are Sal (*Shorea robusta*) followed by Asna *(Terminalia tomentosa)*, Jamun *(Syzygium cumini)*, Karma (*Adina cordifolia*), Amala *(Phyllanthus emblica)*, Harro *(Terminalia chebula)*, Barro (*Terminalia bellirica)*, etc. Other vegetation related informations of the studied community forest are presented in Table 1.

**Table 1:** Detail information about community forest attributes.

Forest attributes	Unit	Values (mean)
Tree growing stock	m^3^ ha^−1^	181.17
Pole growing stock	m^3^ ha^−1^	39.21
Tree density	no. ha^−1^	62
Pole density	no. ha^−1^	339
Sapling density	no. ha^−1^	1897
Seedling density	no. ha^−1^	14012
Tree height	m	19.48
Tree diameter	cm	76.4
Canopy cover	%	70
Soil type	–	loamy soil

### 2.2 Management modality of the community forest

This community forest was established in 1994 just after the established of Forest Act of Nepal, 1993. Irregular shelterwood system under scientific forest management was applied in this community forest from 2020 AD. The management plan for Sal in this forest was structured with an 80-year rotation period and a 10-year regeneration period [9,15]. To facilitate this, the forest was divided into 8 sub-compartments, each further divided into 10 annual working units (each having 3 ha area) (Fig. 1). Each year, one working unit within a sub-compartment is harvested, adhering to a 10-year felling cycle. This means that over a decade, each working unit within a sub-compartment will be managed once. The selection of sub-compartments for felling is based on the growing stock present, ensuring that areas with higher growth are prioritized for sustainable yields. For instance, sub-compartment C1S6 having area 30 ha was chosen due to its superior growing stock compared to others. Data collection during this period revealed that only three working units within C1S6 had been harvested and managed, termed as “managed forest stand” [17]. In contrast, the adjacent three working units where the system was not yet applied were considered as “unmanaged forest stand.” In this community forest, yield regulation by volume was adopted, where 4D (dead, diseased, defected and dying) trees followed by matured trees were harvested in a working unit to meet harvestable volume per year. About 15–25 mother trees per ha were left in managed forest stand for promoting regeneration.

### 2.3 Soil sampling and analysis

This study was carried out in 3 managed working units (forest stands where management interventions were implemented sequentially (i.e., year-wise) starting from 2020 to 2023) and 3 unmanaged working units (unmanaged forest stands as control units) in 2023. We employed a stratified random sampling technique with a sampling intensity of 1.67 %, following the forest inventory guidelines set by the government of Nepal [62]. Random sample plots within each working unit were identified using ArcGIS 10.8. A plot size of 10 m * 10 m was selected for soil sample collection [9,15]. As a result, a total of 30 composite soil samples were collected—15 from managed forest stands and 15 from unmanaged forest stands, from a depth of 0–30 cm. Soil samples were taken from the four corners and the center of each plot using a soil auger and a 3 cm diameter and 30 cm deep core sampler. The sub-samples from each corner and the center of the plot were expected to provide a representative overview of the soil properties within the sample plot. Given that management practices (including tillage), affect only the topsoil and considering the relatively short implementation period (3 years), we assumed that sampling the top 30 cm of soil would sufficiently capture the impact of the irregular shelterwood system [9]. Furthermore, distance of sampling points from tree trunk is important to minimize the influence of tree canopy and roots on soil properties [63]. Therefore, we maintained an average distance of 4 m between the surrounding trees and sampling points in the plot. Finally, these five sub-samples per plot were thoroughly mixed to create a single composite soil sample (approximately 500 g) for each plot. In total, 30 samples from the 0–30 cm depth were collected and stored in air-tight polybags which were sent to Soil and fertilizer testing laboratory in Banke district, Nepal for laboratory analysis. Soil samples were air-dried and sieved through a 2-mm mesh to remove coarse particles. Subsequently, the soil samples were oven-dried at 105 °C until a constant weight was obtained. Then, soil properties were analyzed through methods presented in Table 2.

**Table 2:** Soil properties and methods of analysis.

Soil properties	Method
Bulk Density	Core sampling method [64]
Texture	Hydrometer method [65]
pH	Potentiometry/Glass calomel pH meter [66]
Organic Carbon (OC)	Walkley and Black method [67]
Total Nitrogen (N)	Kjeldahl method [68]
Available Phosphorous (P)	Olsen's and Somers method [69]
Available Potassium (K)	Flame Photometer method [70]

### 2.4 Soil organic carbon (SOC) stock

To quantify the amount of soil organic carbon stored in the soil (ton ha⁻^1^), formula developed by Pearson et al. [71] based on soil depth (cm), bulk density (g cm⁻³), and the organic carbon (%) was employed. The bulk density of soil was determined by dividing the oven dried mass of soil (g) by the volume of soil (cm³).

$$\text{Bulk}\;\text{density}\;\left(g\;{\text{cm}}^{-3}\right)=\frac{\text{Oven}\;\text{dried}\;\text{mass}\;\text{of}\;\text{soil}\;\left(g\right)}{\text{Volume}\;\text{of}\;\text{soil}\;\left(m^{3}\right)}$$

$$\text{SOC}\;\text{stock}\;\left({\text{ton}\;\text{ha}\;}^{-1}\right)=\text{Bulk}\;\text{density}\;\left(g\;{\text{cm}}^{-3}\right)\times \text{soil}\;\text{depth}\;\left(\text{cm}\right)\times \text{organic}\;\text{carbon}\;\left(\%\right)\times 0.1$$

### 2.5 Soil quality assessment

In this study, we opted for the additive system based on common soil parameters method to compute SQI due to its consistent outcomes, a recommendation also made by Abdu et al. [51] for soil quality assessment. The procedure involved three main stages: (i) selection of relevant indicators, (ii) conversion of indicators into scores, and (iii) integration of the scores to create an index [54,72,73]. The SQI interpretation utilized a scoring method outlined in Table 3 [74]. Bajracharya et al. [74] incorporated weight values for NPK in their equation, relying on soil quality ratings provided by NARC [75] (Table 4). The SQI value was calculated using the following formula [51,74].

$$\text{SQI}=[\left(a\times R_{\text{STC}\;}\right)+\left(b\times R_{\text{pH}}\right)+\left(c\times R_{\text{OC}}\right)+\left(d\times R_{\text{NPK}}\right)$$

where,

**Table 3:** Common soil parameters and ranking values for SQI in Nepal.

	Ranking Values				
Parameters	0.2	0.4	0.6	0.8	1
Soil Textural Class	C, S	CL, SC, SiC	Si, LS	L, SiL, SL	SiCL, SC
Soil pH	<4	4–4.9	5–5.9	6–6.4	6.5–7.5
SOC%	<0.5	0.6–1	1.1–2	2.1–4	>4
Fertility (NPK)	Low	Mod Low	Moderate	Mod. High	High
SQR	Very Poor	poor	Fair	Good	Best

Where, C- Clay, Si- Silt, S- Sand, LS- Loamy sand, CL- Clay loam, SiL- Silty loam.

SC- Sandy Clay, SiCL -Silty clay loam, SiL- Silty loam, SiC- Silty Clay, SL- Sandy loam, SCL- Sandy Clay loam, LS- Loamy Sand, SQR- Soil Quality Rating.

**Table 4:** N, P and K interpretation of soil of Nepal.

**Total N (%)**		Available P (kg ha^−1^)		Available K (kg ha^−1^)	
**Range**	level	Range	level	Range	**level**
**<0.1**	Low	<31	Low	<110	**Low**
**0.1–0.2**	Medium	31–55	Medium	110–280	**Medium**
**>0.2**	**High**	**>55**	**High**	**>280**	**High**

SQI = Soil Quality Index.

R_STC_ = assigned ranking values for soil textural class.

R_pH_ = assigned ranking value for soil pH.

R_OC_ = assigned ranking value for soil organic carbon, RN = assigned ranking value for nitrogen,R_P_ = assigned ranking values, for phosphorus.

R_K_ = assigned ranking value for potassium.

And a = 0.2, b = 0.1, c = 0.4, and d = 0.3 are the weighted values corresponding to each of the parameters.

### 2.6 Statistical analysis

Statistical analysis of all the data was conducted using Microsoft Excel 2010 and R Studio version 4.2.3. Prior to the analysis, normality tests (p > 0.05) were applied, confirming that the data exhibited a normal distribution. To evaluate differences at 0.05 significance level among SOC stock and other soil parameters within each forest type, the Welch *t*-test was employed. Pearson correlation analysis was conducted to identify relationship among measured soil indicators**.**

## 3 Results

### 3.1 General soil properties in managed and unmanaged forest stands

**Fig. 2:**
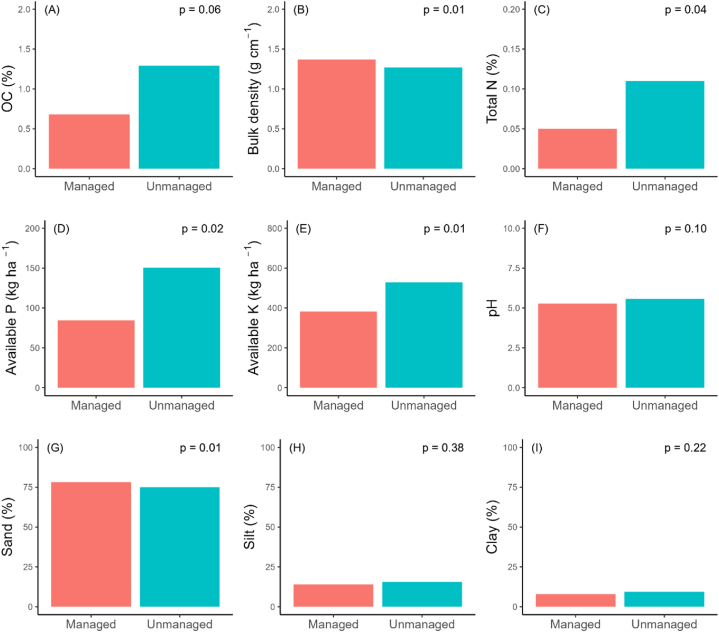
Comparison of soil properties between managed and unmanaged forest stands.

A significantly higher SOC observed in unmanaged forest stand (48.87 ± 1.34 t ha^−1^) than managed forest stand (27.76 ± 1.27 t ha^−1^) (Fig. 2A). However, bulk density was significantly higher in managed forest stand (1.37 ± 0.01 g cm⁻³) than unmanaged forest stand (1.27 ± 0.01 g cm⁻³) (Fig. 2B). Similar to SOC, significantly higher total N recorded in unmanaged forest stand (0.11 % ± 0.03 %) than managed forest stands (0.05 ± 0.00 %) (Fig. 2C). Similar trend was observed for available P i.e. 150.57 ± 26.5 kg ha⁻^1^ in unmanaged forest stands and 84.46 ± 4.63 kg ha⁻^1^ in managed forest stands (Fig. 2D) and available K i.e. 529.31 ± 48.19 kg ha⁻^1^ in unmanaged forest stands and 381.8 ± 25.16 kg ha⁻^1^ in managed forest stands (Fig. 2E). No significant differences on pH was observed between managed and unmanaged forest stands (Fig. 2F). In unmanaged forest stands, soil fractions consisted of 75.08 % sand, 15.55 % silt, and 9.37 % clay, while managed forest stands showed 78.19 % sand, 13.96 % silt, and 7.85 % clay (Fig. 2G, H, 2I).

**Fig. 3:**
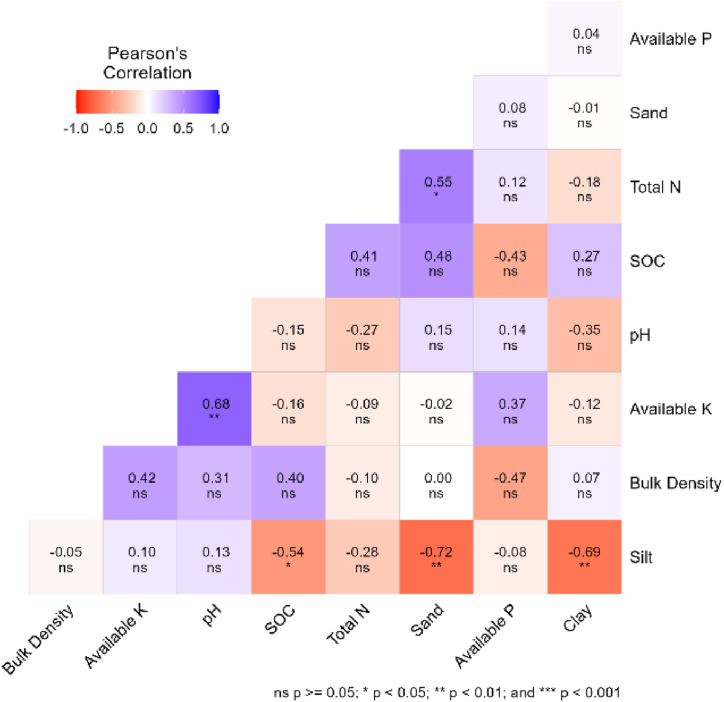
Correlation heatmap of soil properties in managed forest stands.

**Fig. 4:**
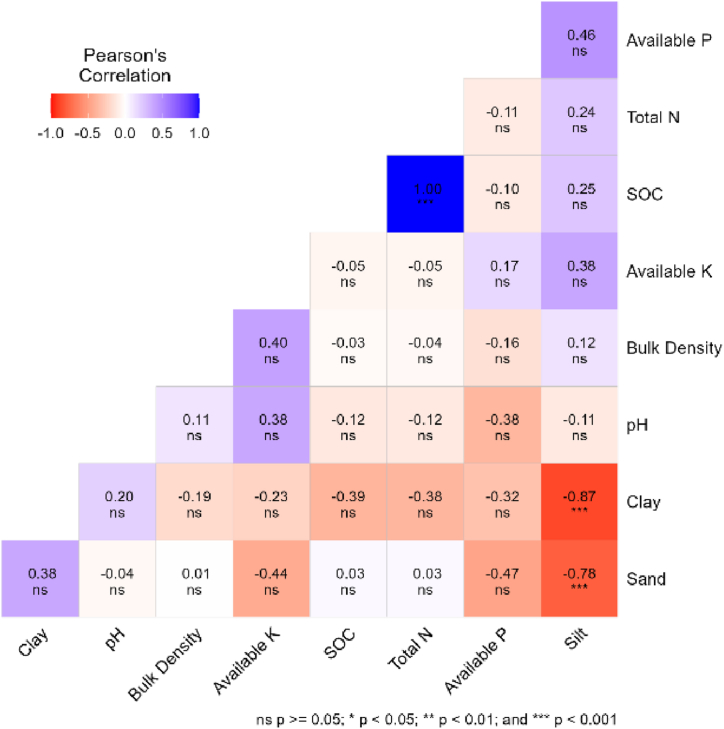
Correlation heatmap of soil properties in unmanaged forest stands.

In managed forest stands, significant positive correlation (r = 0.68, p < 0.01) was observed between available K and pH, indicating a moderate-strength association (Fig. 3). Furthermore, a statistically significant negative correlation (r = −0.54, p < 0.05) was identified between SOC content and silt, signifying a moderate-strength inverse relationship. The negative correlation of −0.72 (p < 0.05) between sand and silt and −0.69 (p < 0.01) between silt and clay highlights strong negative associations. Lastly, a significant positive correlation (r = 0.55, p < 0.05) between sand and Total N suggests a moderately strong positive relationship. Similarly, in unmanaged forest stand, a perfect positive correlation (r = 1.00, p < 0.001) is observed between SOC content and Total N, indicating a strong and direct relationship between these two crucial components (Fig. 4). Additionally, a highly significant negative correlation (r = −0.87, p < 0.001) exists between clay and silt fractions. Furthermore, a significantly negative correlation (r = −0.78, p < 0.001) is identified between sand and silt fractions.

### 3.2 SOC stock in managed and unmanaged unit type

**Fig. 5:**
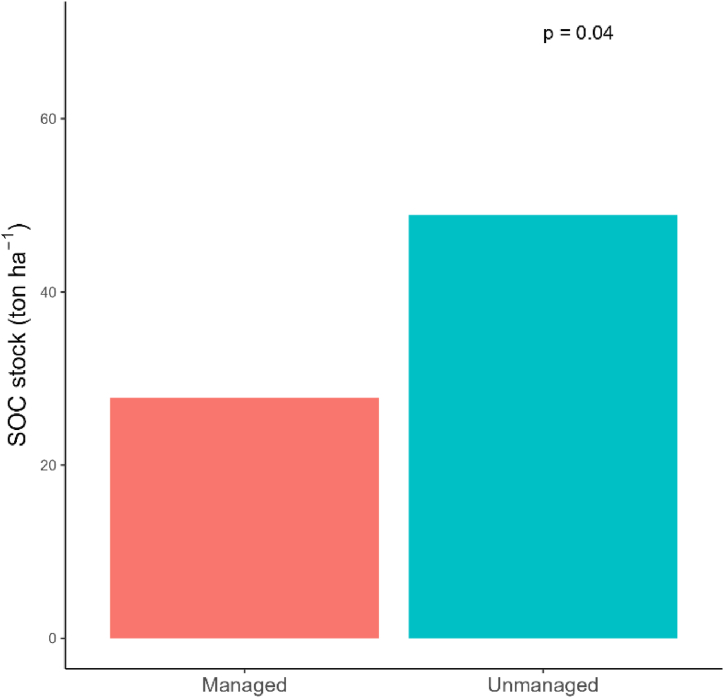
SOC stock of managed and unmanaged forest stands.

Our study found significant difference in SOC stock between unit types (p < 0.05). Higher SOC stock was observed in unmanaged forest stands (48.87 ± 1.34 ton ha^−1^) than managed forest stands (27.76 ± 1.27 ton ha^−1^) (Fig. 5).

### 3.3 SQI of managed and unmanaged unit type

**Fig. 6:**
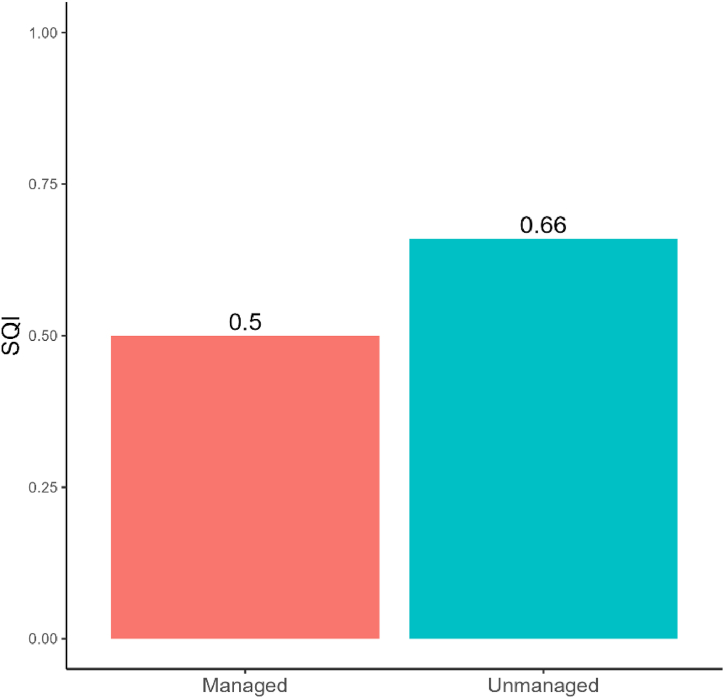
SQI ranking of managed and unmanaged forest stands.

SQI value was 0.66 which falls under good category in unmanaged forest stand. However, SQI values was 0.50 belonging to fair category in managed forest stands (Fig. 6).

## 4 Discussion

### 4.1 General soil properties in managed and unmanaged forest stands

Our study observed negative effect of application of irregular shelterwood system on soil properties which aligns with Aryal et al. [9] in Lumbini collaborative forest in the central lowlands of Nepal. Lower soil carbon was reported in managed forest stands (Fig. 2A) which could be due to intensive biomass harvesting leading to reduced organic matter input [76]. Higher soil bulk density in managed forest stands (Fig. 2B), likely to be resulting from the use of conventional machinery and concentrated logging activities [9]. Spinelli et al. [77] found that conventional logging practices affect more than 50 % of the surface soil as well as regeneration and standing trees. Higher bulk density can lead to reduced soil aeration, restricted root growth, lower water infiltration, decreased fertility, and impaired soil structure which is detrimental to soil health, forest regeneration, and productivity [78,79]. Soil nutrients such as N, P and K were also observed lower in managed forest stands (Fig. 2C, D, 2E). The lower N content could be due to removal of legumes from soil along with other vegetation due to species focused management activities (*S. robusta* in case of studied forest) [9]. Forest management system in Nepal are focused on retaining the most valuable species i.e. *S. robusta* which was found in previous studies that assessed species diversity under irregular shelterwood system in Nepal [9,17,19,60,61]. This species specific management with removal of legumes for creating future stands of single (i.e., *S. robusta*) species could disrupt the natural nitrogen-fixing processes provided by legumes, leading to decreased soil nitrogen levels. Similarly, removal of tree in irregular shelterwood system could affect nutrient recycling which could lower the soil nutrients [27,80]. This effect is further exacerbated by leaching and runoff through an open canopy, which can further lower soil nutrient levels [9]. Furthermore, removing tree cover can alter soil pH (Fig. 2F) and chemistry, affecting nutrient solubility and availability. This impacts nutrient absorption by plants and reduces overall soil nutrient levels. Therefore, the removal of trees not only reduces the direct contribution of nutrients through organic matter but also impacts the overall nutrient availability in the soil [27,80]. We also observed high sand content and low clay content in soil of managed forest stands (Fig. 2G, H, 2I). This can lead to reduced water retention and faster drainage which can further cause nutrient leaching, lower soil fertility, and increased erosion [81]. Forest management practices can directly or indirectly affect both the physical and chemical properties of soil and influence forest productivity [45]. A well-designed forest management strategy is crucial for preserving soil quality and function [82]. Its effectiveness, however, relies on factors such as the intensity and duration of soil disturbances, the timing of interventions, the extent of tree felling, and the methods used for site treatment [83,84].

### 4.2 SOC stock in managed and unmanaged forest stands

Our study observed lower SOC stock in managed forest stands than that of the unmanaged forest stands (Fig. 5). This may be due to the reduced density of large trees and their foliage, which are significant sources of litter and organic matter that contribute to SOC [9]. Furthermore, excessive sunlight on the forest floor due to canopy opening in irregular shelterwood system can increase soil temperatures, leading to the oxidation of soil carbon into carbon dioxide and consequently lowering SOC levels [85]. This aligns with Aryal et al. [9] which also reported lower value of SOC in managed blocks (30.29 ton ha^−1^) than unmanaged one (38.41 ton ha^−1^). Likewise, our SOC stock assessment is consistent with the national evaluation by the Department of Forest Research and Survey, Nepal, in 2014, which found the average SOC in Nepal's lowland forests to be 33.66 tons/ha [3]. However, SOC stock in managed forest stands is lower than national average. Several studies also supports our findings of lower SOC stock in relatively undisturbed forests due to increased litter production, faster decay rates, and greater populations of bacteria and fungi [[86], [87], [88]]. Additionally, a global review by Noormets et al. [89] found that managed forests have approximately 50 % lower carbon stocks than unmanaged forests. Intensive biomass harvesting could led to short term significant decreases in SOC stock in the forest floor [76]. However, the long-term impact of intensified forest harvesting on SOC stocks in is not currently clear, and long-term experiments are needed. Clarke et al. [90] have also suggested a need to investigate long term impact of intense forest harvesting. Since the scientific forest management program in Nepal has been established to support national REDD + goals, which aim to maintain forest and soil carbon content [9], it is essential to evaluate both its short-term and long-term effects on soil carbon.

### 4.3 SQI in managed and unmanaged forest stands

Our study demonstrated notable disparities in soil quality between the unmanaged and managed forest stands (Fig. 6). The unmanaged forest stand exhibited a higher soil quality ranking, marked as “good” at 0.66, while the managed unit received a “fair” rating at 0.50. The lower soil quality in managed forest stands could be attributed to limited litter accumulation and reduced crown density due to the harvesting of trees [50,91]. Trees play a crucial role in nutrient cycling within a forest ecosystem. When trees are removed, the cycle of nutrient uptake and return through litter fall is disrupted, leading to a decrease in soil nutrient levels over time [27,80]. Extensive forest management activities alter soil physical, chemical, and biological properties, causing reduced productivity due to surface erosion, mass flow, soil compaction, and rutting and puddling [92]. However, it is important to note that our research was conducted only three years after management practices were initiated. Therefore, the observed decline in soil quality might be a short-term result. Soil organic matter may decrease as a result of reduced litter fall, leading to lower nutrient levels such as nitrogen, phosphorus, and potassium [27]. The structure of the soil can also degrade due to compaction from harvesting machinery and reduced root biomass [93]. Additionally, soil moisture retention may decline as the diminished canopy cover increases evaporation rates [94]. Soil microbial activity can decrease due to the disturbance and reduced organic inputs, while soil pH may temporarily alter due to changes in organic matter and nutrient cycling [95,96]. Over a longer period, with continued proper management practices and natural regeneration, the forest ecosystem is likely to recover, and soil quality may improve. The irregular shelterwood system may initially reduce tree density, but it promotes natural regeneration and growing stock by decreasing competition [17]. As vegetation regrows, litter accumulation increases, which enhances soil organic matter, nutrient levels, and microbial activity. Root growth and organic inputs will help restore soil structure, while the developing canopy cover will aid in retaining soil moisture. Additionally, soil pH is likely to stabilize as organic matter and microbial processes normalize. Ultimately, sustainable forest management aim to maintain and enhance the quality of the soil resource base while ensuring non-declining or positive forest productivity trends [97].

## 5 Conclusion

Understanding the impact of irregular shelterwood system on soil is crucial for effective forest management. Aligning with our initial hypothesis, our study reported negative impact on soil carbon stock and soil quality in Sal forest due to the application of the irregular shelterwood system. We observed greater SOC stock and higher SQI value in unmanaged forest stands than managed forest stands. However, further research encompassing different soil depths and management regimes is needed to validate this result and draw concrete conclusion. Our study focused on soil properties up to 30 cm depth within a community forest management regime. Future research should investigate soil depths up to 1 m within community forests to enhance understanding of SOC stock and soil quality impacts. Future studies should also explore impact of irregular shelterwood system in different forest management regimes beyond community forests. Furthermore, longitudinal research studies should be conducted to better understand the long-term impacts, since this study was conducted just three years after management began. This will help forest managers to develop more targeted and effective management strategies for sustainable forest management.

## Funding

This research was supported by the 10.13039/100011219Ministry of Forests, Environment and Soil Conservation, Lumbini Province, Nepal as a B.Sc. Thesis research grant (Grant No: 32900012) to Mr. Anil Poudel in 2023.

## Data availability statement

The data will be made available on request.

## CRediT authorship contribution statement

**Anil Poudel:** Writing – original draft, Investigation, Funding acquisition. **Santosh Ayer:** Writing – review & editing, Writing – original draft, Visualization, Methodology, Formal analysis, Data curation, Conceptualization. **Rajeev Joshi:** Writing – review & editing. **Jeetendra Gautam:** Supervision, Formal analysis. **Sachin Timilsina:** Writing – review & editing. **Keshav Khadka:** Supervision. **Kishor Prasad Bhatta:** Writing – review & editing. **Menuka Maharjan:** Writing – review & editing.

## Declaration of competing interest

The authors declare that they have no known competing financial interests or personal relationships that could have appeared to influence the work reported in this paper.
